# 

**Published:** 1829-03

**Authors:** 


### *TRANSYLVANIA UNIVERSITY.*

21. At a public commencement held in the Chapel of the University, on Tuesday the 10th of March, 1829, the degree of Doctor of Medicine was conferred on the following gentlemen, alumni of the School, who had undergone satisfactory examinations before the Medical Faculty, Trustees and President, and written Theses on subjects annexed to their names.

### *FROM KENTUCKY.*

Robert Stuart Apperson, of Richmond *on Sarcocele.*

John Charlton Beatty, of Mason C. *on Paracyesis Abortus.*

Richard William Ferguson, of Louisville, *on the Physiology and Pathology of the Teeth.*

Charles Hay, of Fayette County, *on the Anatomy and Physiology of the Stomach.*

Henry Hopson, of Christian County, *on Menstruation.*

John Terrel Lewis, of Lexington *on Milk Sickness.*

Preston Lindsay, of Scott County, *on the Identity of Summer, Fall, and Winter Epidemics.*

Thomas Jefferson Moore, of Bowlinggreen, *on Erysipelas.*

Alexander Hamilton Peck, of Lexington, *Phthisis Pulmonalis.*

James Ritchie, of Lexington, *on Hydrocele.*

Thomas Stevenson, of Meade County, *on Syphilitic and Pseudo-syphilitic affections.*

Robert James Waggoner, of Columbia, *on the Functions of the large Intestines.*

Henry Francis Washington, of Russelville, *on Dyspepsia.*

Walter Carr Winter, of Lexington, *on Ophthalmia purulonta.*

### *MISSISSIPPI.*

Charles W. Harris, of Pike C. *on Lacteal and Venous Absorbtion.*

Charles Shaw, of Jefferson County, *on the Nature and Treatment of Syphilis.*

Solomon Tracy, of Meadville, *on Uterine Hœmorrhage.*

William Walworth Usher, of Wilkinson County, *on Malignant Congestive Bilious Fever.*

William Kinne Wilson, of Adams County, *on Dropsy.*

Elis Pusey Passmore, of Meadville, *on Uterine Hœmorrhage.*

### *ALABAMA.*

William Kelsay Adams, of Limestone County, *on the Medical Topography and Endemic Fever of Limestone County, Ala.*

Richard Tucker Harper, of Lawrence County, *on the Analogy between the Liver and the Brain.*

Jacob Chancy Jordan, of Linden, *on the Operation of Lithotomy.*

Sidney Smith Prince, of Lagrange, *on the Medical Topography and Endemic Fever of Franklin Co. Ala.*

William Williams Wilson, of Monroe County, *on Sleep.*

### *TENNESSEE.*

Erasmus Darwin Fenner, of Jackson, *on the Uterine Hœmorrhage of Pregnancy.*

James McKinney Grey, of Sumner County, *on Opium.*

John McCall, of Smith County, *on Purgative Medicines.*

Henry Holmes Treadway, of Murfreesborough, *on Syphilis.*

### *SOUTH CAROLINA.*

Archibald Ingram Barron, of York District, *on Syphilis.*

Thomas Latta Dunlap, of Lancasterville, *on Hernia of the Abdominal Rings.*

Gilbert Tennent, of Abberville District, *on the Medical Topography of Abbeville District, S. C. with Observations on Bilious Fever, as it occurred there in the Summer of* 1828.

FRANKLIN WILLIAMS, of Laurens District, *on Diet.*

### *VIRGINIA.*

WILLIAM BOMAR, of Halifax County, *on Hydrocele.*
PETER LEATH PENN, of Patrick County, *on Curved Spine.*

### *LOUISIANA.*

OWEN CONNELLY BLOUNT, of East Baten-Rouge, *on the Teeth.*
WILLIAM HENRY LYNE, of East Feliciana, *on Bilious Fever.*

### *MICHIGAN.*

RICE MCCOY, of Carey's Station, *on the Actual Cautery.*
JOSEPHUS MCCOY, of Carey's Station, *on the Pathology and Medical Treatment of Calculous Affections.*

### *OHIO.*

RESIN THOMPSON, of Harrison County, *on Digestion.*

### *MEDICAL COLLEGE OF OHIO.*

22. The Commencement for conferring degrees in this Institution, was held in the College Edifice on the 15th inst. The following is a list of the graduates, with the subjects of their respective Theses:

*David Baird*, of Middletown, O. on Ascites.
*Milton Dunlap*, of Ripley, O. on Dependence of Function.
*Benjamin Hegeman*, of Lebanon, O. on Anatomy and Physiology as the asis of Correct Medical Knowledge.
*Lachlan M. Johnson*, of Cincinnati, O. on Yellow Fever.
*Abraham T. Markle*, of Steubenville, O. on Causes, Symptoms and Treatment of Intermittents.
*Sydney S. Alexander*, Shepherdsville, Ky. on Rubeola Vulgaris.
*James Morrison*, Bourbon county Ky. on Secale Cornutum.
*Zacharia C. Offut*, Georgetown, Ky. on Cholera Infantum.
*David M. Pollard*, Shelby county, Ky. on Delirium Tremens.
*William H. Robards*, Elizabeth, Ky. on Dyspepsia.
*Joseph Somes*, Glasgow, Ky. on Datura Stramonium.
*William T. Thornton*, Oldham county, Ky. on Dysentery.
*John H. F. Thornton*, Oldham county, Ky. on Hydrocephalus.
*Job M. Baker*, Vicksburgh, Mississippi, on Cynanche Trachealis.
*Joseph W. Hegeman*, Vicksburgh, Mississippi, on Bilious Fever.
*William Monett*, Washington, Mississippi, on Dengue.
*Archibald Smith*, Amity county, Mississippi, on Pneumonia Biliosa.
*William W. Bunnel*, Washington, Ind. on Sick Stomach, or *Milk Sickness.*
*James C. Kelso*, Livonia, Ind. on Epidemic Dysentery.
*John Lee Rogers*, Greenville, Illinois, on Abortion.
*Samuel Lessley*, Abbeville, S. Carolina, on Functional Derangements of the Liver.

### INDEX TO VOLUME SECOND.

A.

Abdominal Ring, . 528
Absorbent vessels and glands, 540
Acetate of Ammonia in uterine diseases, . , 649
Acupuncturation, . 369—371
Alcohol, its origin, . . 12
composition, . . 88
proportion in different beverages, . . . 88
Amputation without ligatures, 55
of the lower jaw, . 212—468
Mr. Lizars' method, 435
circular and flap operations, 650
Angina suffocans, . . 473
Animal tissues, structure of, 596
Ardent Spirits, nature of . 11
effects, . . . 12
necessity for . . 14
causes of their abuse, . 21
morbid effects of . . 61
desolating, do. . . 71
restrictions on their use, 77
prize dissertation on . 389
an address on, . 389
Arteries, obstruction in . 370

B.

Balsam Copaiva, eruptions caused by, . . . . 60
Biography of Dr. Thomas Hinde, . . 625
Birds, American, engravings of, . . . 221
Bladder, irritable, . 496
Blisters, to prevent abortion, 384
in Measles, . . 365
Blood in the veins and capillary vessels, difference of 639
coagulation of . 642
Board of Health, in Cincinnati, . . . 221
Bones, huge fossil, . 159
Botany, books upon,
its study commended, 205
Brain, memoir upon, . 418
Brucia, . . . 39
Burns, . . . 495

C.

Calculi Urinary, in an Ox, 450
analysis of, . . 451
their physical characters, 573
their ætiology, . . 575
ages most liable to them, 575
Capillary vessels, dilatation of, . . . . 268
Carbuncle, . . . 483
Cataract, double knife for, 231
Catheters, straight, . 577
rules for their introduction, 577
Cephalo-spinal fluid, . 419
Coffee, . . 381
Cinchonia, . . . 45
Cincinnati, Weather and Diseases of, 116—184—216
Chorea, hydrocyanite of iron in, 650
Colchicum Autumnale, 94
Cold air, inhalation of in pulmonary diseases, . 383
Colica pictonum, . 369
Collapse of the system, 413
Combustion, spontaneous, of the human body, . 130
Congestion, on the doctrine of, . . 396
venous, . 400
Constitutional origin of local diseases, . 459—472
Convention, medical, writ for, . . 213
Cornea, opacity of, . 646
Crucifereæ, . . 198
Cupping in poisoned wounds, 109
in the vaccine disease, 378
Cyanide of Potassium, . 97
of Zinc, . . 98
of Iodine, . . 98

D.

Delphinia, . . 99
Deltoid muscle, . . 531
Dengue, . . 363—428
Dislocations, 515, 516, 518, 519, 520, 521, 522, 525, 526
Dropsies, treatment of, 636
Drowning, resuscitation from, 499

Drowning, signs of, 647

E.

Electricity, . . 479
Emeta, . . 44
Empyema, cases of, . 612
Endermic medication, . 173
Epidemic constitutions, 553
Epilepsy from bathing, . 454
hydrocyanate of iron in, 650
Epiploon, disease of, . 373
Ergot in hydatids of the uterus, . . 181
Erysipelas, . 482
Excitement, suffocated, 409
Expansibility, a vital property of the capillaries, . 258
Expectoration, poisonous, 277
Eye Infirmary, Cincinnati, 394
annual report of, . 603
Eye, inflammation of, 645

F.

Fever, pathology of 396—503, 608
Autumnal 444—552—564
its local origin, . 465
Sympathetic inflammatory 466
Hectic, . . 466
Sympathetic, irritative, 467
observations on, by Medicus, No. 1. . . 503
No. 2. . . 607
Fifth pair of nerves, functions of illustrated, . 598—600
Fœtal circulation, . 209
Formulary for the preparation of new medicines, 35—92
Fractures, 376, 492, 514, 518, 519, 520, 521, 522, 526

G.

Galvanism, effects of, 602
Gentiania, . 99
Godman Dr. resignation of, 278
Graduates in medicine, catalogue of, . 652,654

H.

Hæmorrhage, uterine, practical observations on, 241
from wounds and ulcers, 492
Heart, peculiar disease of, 168
gunshot wound of, . 330
wounds of, . 333
softening of, . . 337
sympathetic affections of, 358
Herbarium, directions for forming . . , 207
Hesperideæ, . . 198
Hip joint diseases of, . 523
Holyoke Dr. tribute of respect to, . . 278
Hydatids of the uterus cured by Ergot, . 181
Hydrocyanic acid, . 94
a cause of autumnal fever, 564
Hydrocyanite of potash, 97
Hydriodate of potash, ointment of, . . 105, 392
Hydrophobia, . . 497

I.

Ice in puerperal peritonitis, 210
Inflammation, . 474
phlegmonous, . 474
chronic, . . 477
irritative, . . 481
erysipelatous, . 482
of the eye, . 645
Insanity, puerperal, 638
Intellection, views of, 546
Intemperance, Discourse on, 11
Intestinal investigations, 171
Iodine, 100, 121, 392, 640
Iritis cured by Iodine, 293
Irritation of a particular organ, . . . 403
constitutional, . 406, 469
Ischuria, cured by the cold affusion, . 177
Issues, . . 480

K.

Kidneys, ulceration of, 290
extirpation of, . 376
diseases of, . 642
Knee joint, removal of loose substances from, . 648

L.

Labiateæ, . . 202
Laryngitis, . . 373
Lead, plates of, in ulcers, 371
Lectures, by Mr. Abernethy, 458
western medical, 223–4
Life, nature of, . . 545
Ligatures in intermittents, 645
Lip, upper, enlargement of, 375
Liver, physiology of, . 534
morbid anatomy of, . 535

Lithontrity, . . 570
history of, . . 576
apparatus for, . . 578
practice of, . . 582
in females, . . 586
objections to, . 586
table of cases, . . 588
Lithotomy compared with
lithontrity, . . 594
Lungs, wounds of, . 329
emphysema of, . 341
irritability of their air cells, 536
morbid structure of, . 539

M.

Magnoliaceæ, . . 196
Marsh miasma, . . 553
Medical classes, western, 506
Mercury, its absorption, 212, 636
Meteoration, sensible, . 553
Mineral waters, . 142
rules to be observed in
visiting them, . . 143
of Harrodsburgh, . 145
of Rochester, . . 151
of the Olympian valley, 151
of the Blue Licks, . 156
of Big Bone, . . 158
of the Yellow spring, 164
Morphia, . . . 41
Mortification, . . 483
Muscular motion lost, . 599

N.

Nævi materni, ligature in, 368
Narcotin, . 41
Necrology, 64, 277, 394, 606
Nerves, natural system of, 307
pneumogastric division of, 377
Neuralgic affection, . 623
effects of galvanism on, 602
Nux vomica, . . 37

O.

Opium, . . . 41
death from its external use, 215
Orbicularis palpebrarum, 531

P.

Pancreas, morbid structure of, 542
Paracentesis thoracis, 612
Patella, fractures of, 250
Pericarditis, 356, 359, 361, 537
Placenta, communication
between it and the uterus, 209
Plants, their medical proper-
ties and affinities, 183
natural orders of, 203
Poisoned wounds,
treatment of, 109, 214, 386
Poisoning with sulphuric
acid, . . 114
by opium, . . 179
Peritonitis, puerperal, 210
Pressure in poisoned
wounds, . 109, 386
in ununited fractures, 114, 274
Prussic acid, . 94
Pulmonary veins, valves of, 596
Pylorus, rupture of, 126

Q.

Quina, . . 45

R.

Resuscitation from drowning, 499
Rheumatic inflammation of
the heart, diagnosis of, 58
Ruptures . . 528

S.

Salivation, from inhaling the
breath of a patient with
ptyalism, . . . 276
Scalds, . . 495
Sensation and motion, con-
genital absence of, . . 211
Sensation, views of, 546
Sensibility, impaired, 599
Solania, . . . 98
Spina bifida, . . 650
Spine, diseases of, 497
Spleen, enlargement of . 293
physiology of, . 543
Stomach, physiology of, 532
morbid anatomy of, 533
Strichnia, . . 37
Sympathy, cause of, . 547

T.

Tetanus, . 211, 470, 738
Tic Douloureux, . 490
Trephining, . . 541
Tonsils, excision of, . 59

U.

Ulcers cured by plates of
lead, . . 371
treatment of, . 485
varicose, . . 489
Umbelliferææ, . . 200

Ureters, disease of 642

Urethra, its anatomy 577

Uterine diseases, acetate of ammonia in, . 649

Uterus, extirpation of, 640

V.

Vaccination, test of its efficacy, . . 113
Veratria, . . . 92
Violaceæ, . . 198
Viscera, abdominal and thoracic, reciprocal influences of, . . . 281
Vision after a læsion of the optic nerves . 601

W.

White mustard seed, 50
White Swellings, . 640
Wound, gunshot, of the lungs and heart, . 329
Wry neck, . . 529

